# Engineered Inner Ear Drug Delivery Systems for Hearing Loss Treatment

**DOI:** 10.34133/research.1396

**Published:** 2026-08-21

**Authors:** Zhiyan Xuan, Xiayidan Maimaitikelimu, Jie Zhan, Minli Li, Jingjing Gan, Huan Wang

**Affiliations:** ^1^ Biological Laboratory of Hetao Cooperation Zone, The Eighth Affiliated Hospital of Sun Yat-sen University, Shenzhen 518033, China.; ^2^ School of Biological Science and Medical Engineering, Southeast University, Nanjing 210096, China.; ^3^ Department of Rheumatology and Immunology, Nanjing Drum Tower Hospital, School of Biological Science and Medical Engineering, Southeast University, Nanjing 210096, China.

## Abstract

Hearing loss is becoming a common disease worldwide, affecting individuals across all age groups, from infants to older adults. However, the unique anatomical barriers of the inner ear limit the effectiveness of conventional systemic therapies, highlighting the need for advanced drug delivery strategies. Engineered inner ear drug delivery systems have emerged as promising approaches for improving drug targeting, retention, and therapeutic efficacy. In this review, we provide a comprehensive overview of the advances by integrating 3 major aspects: major administration routes, diverse therapeutic agents, and delivery carriers employed in inner ear treatment. We first introduce systemic administration, trans-tympanic drug delivery, intracochlear drug delivery, and cochlear implantation (CI). We then summarize anti-inflammatory drugs and antioxidants for the treatment of inner ear disorders. Subsequently, we discuss multiple nanocarriers, viral vectors, injectable hydrogels, and other innovative carriers used in inner ear drug delivery, highlighting their design principles and therapeutic applications. We also illustrate recent progress in clinical trials and discuss the difficulties associated with translating experimental systems into clinical applications. Finally, we outline current limitations and future opportunities, together with potential strategies to address these challenges.

## Introduction

Hearing loss is gradually becoming a global problem. Noise pollution, infection, ototoxic drugs, and genetic factors contribute to disease progression, causing important impairments in patients. These pathological factors can damage the structures responsible for sound transmission or disrupt the transmission of neural impulses to the brain, thereby resulting in conductive hearing loss, sensorineural hearing loss (SNHL), or mixed types. The associated difficulties in communication may cause long-term social isolation and aggravate cognitive disorders, while challenges in securing and maintaining employment further increase the financial burden on patients, their families, and society as a whole [1]. Therefore, hearing loss represents a substantial burden from both individual and societal perspectives.

Based on these pathological mechanisms, therapeutic strategies mainly target the restoration or protection of cochlear structures and neural components. Currently, cochlear implantation (CI) is an effective intervention when hearing aids are no longer beneficial, as it can convert sound into electrical signals and directly stimulate spiral ganglion (SG) cells of the cochlear nerve [1,2]. However, whether a patient’s condition is suitable for CI must be carefully evaluated. To achieve pharmacological intervention at specific targets, multiple drug delivery routes have been explored. Systemic administration is widely used but often limited by low drug solubility, poor bioavailability, and the presence of the blood–labyrinth barrier (BLB), which restricts drug penetration into the inner ear [3]. To overcome these limitations, local delivery strategies such as trans-tympanic membrane (TM) delivery and intracochlear administration have been developed, enabling more direct access to the inner ear and improving targeting efficiency. With the advancement of therapeutic approaches, a variety of therapeutic agents including antioxidants and anti-inflammatory agents have been reported for hearing loss treatment. Moreover, gene therapy has made important progress over the past few years, particularly in the treatment of otoferlin (OTOF)-related hearing loss [4,5]. To date, several approaches have been demonstrated to be effective in animal models [6,7]. Multiple clinical trials of OTOF gene therapy are currently recruiting participants (NCT05821959, NCT06722170). Nevertheless, the complex anatomy of the inner ear necessitates the development of suitable delivery systems to enhance therapeutic efficacy.

**Fig. 1.**
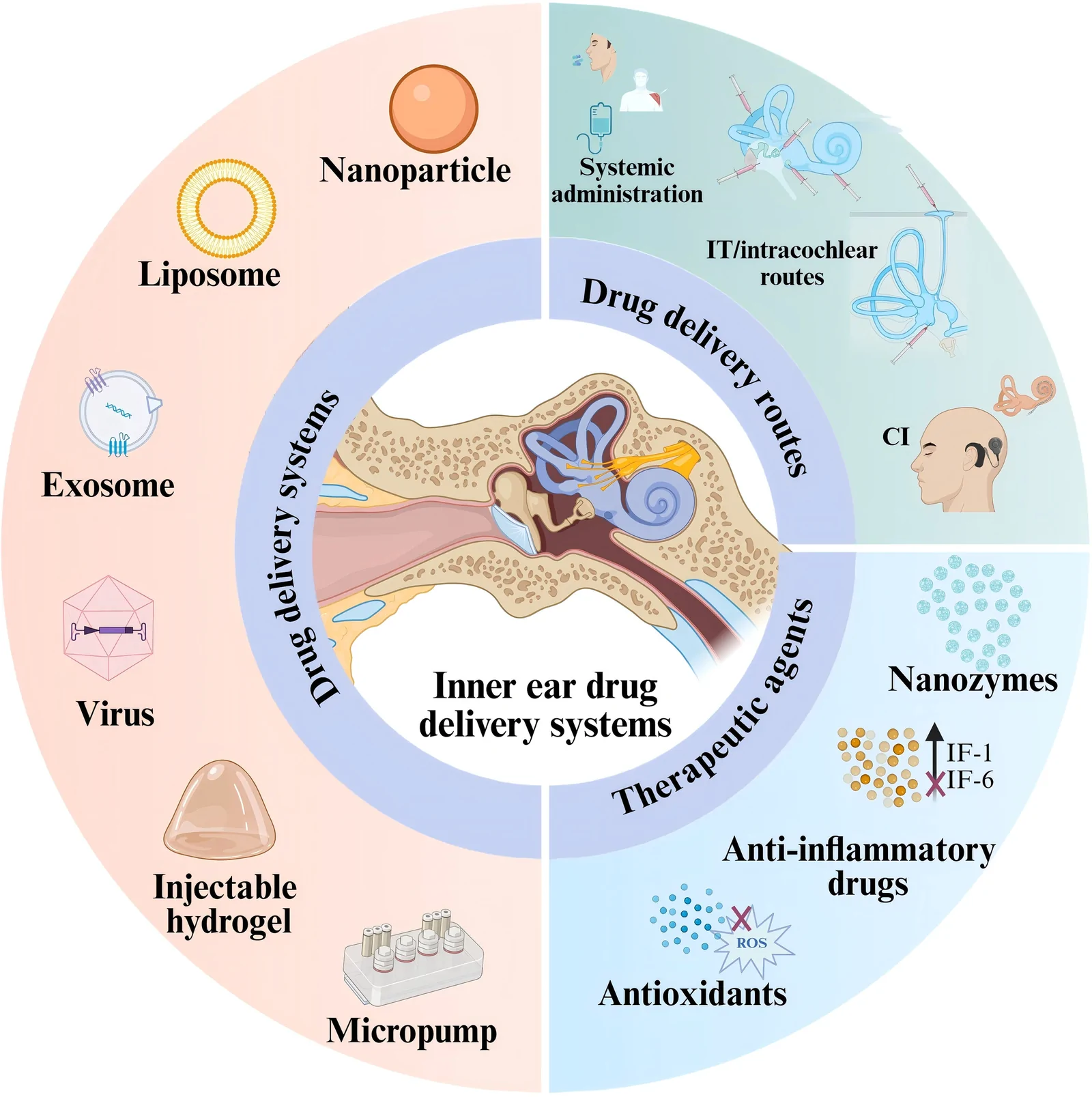
Overview of inner ear drug delivery systems. (Figure was created with BioRender.com.) IT, intratympanic injection; CI, cochlear implantation.

This review outlines the development of inner ear drug delivery systems for hearing loss treatment (Fig. 1). First, we introduce the physiological structures of the ear and emphasize the need for engineered inner ear drug delivery strategies. Next, we systematically summarize current drug delivery routes for inner ear treatment, including systemic administration, TM drug delivery, intratympanic drug delivery, and intracochlear drug delivery. We then discuss current therapeutic agents and drug delivery carriers for hearing loss therapy and illustrate recent advances regarding ongoing clinical trials. Finally, we address the challenges and limitations of the current inner ear drug delivery systems and point out possible solutions. Overall, rather than focusing on specific aspects such as biological barriers, biomaterial applications, or introducing delivery routes alongside corresponding carrier systems, our review provides a dedicated and integrated discussion [8–10]. Generally, this structured framework provides a systematic and comprehensive perspective, which facilitates a clearer and more holistic understanding of engineered inner ear drug delivery systems. This review is expected to inspire researchers in related fields and provide them with theoretical support.

## Ear Structure and Hearing Loss

### Physiological structure of ear

**Fig. 2.**
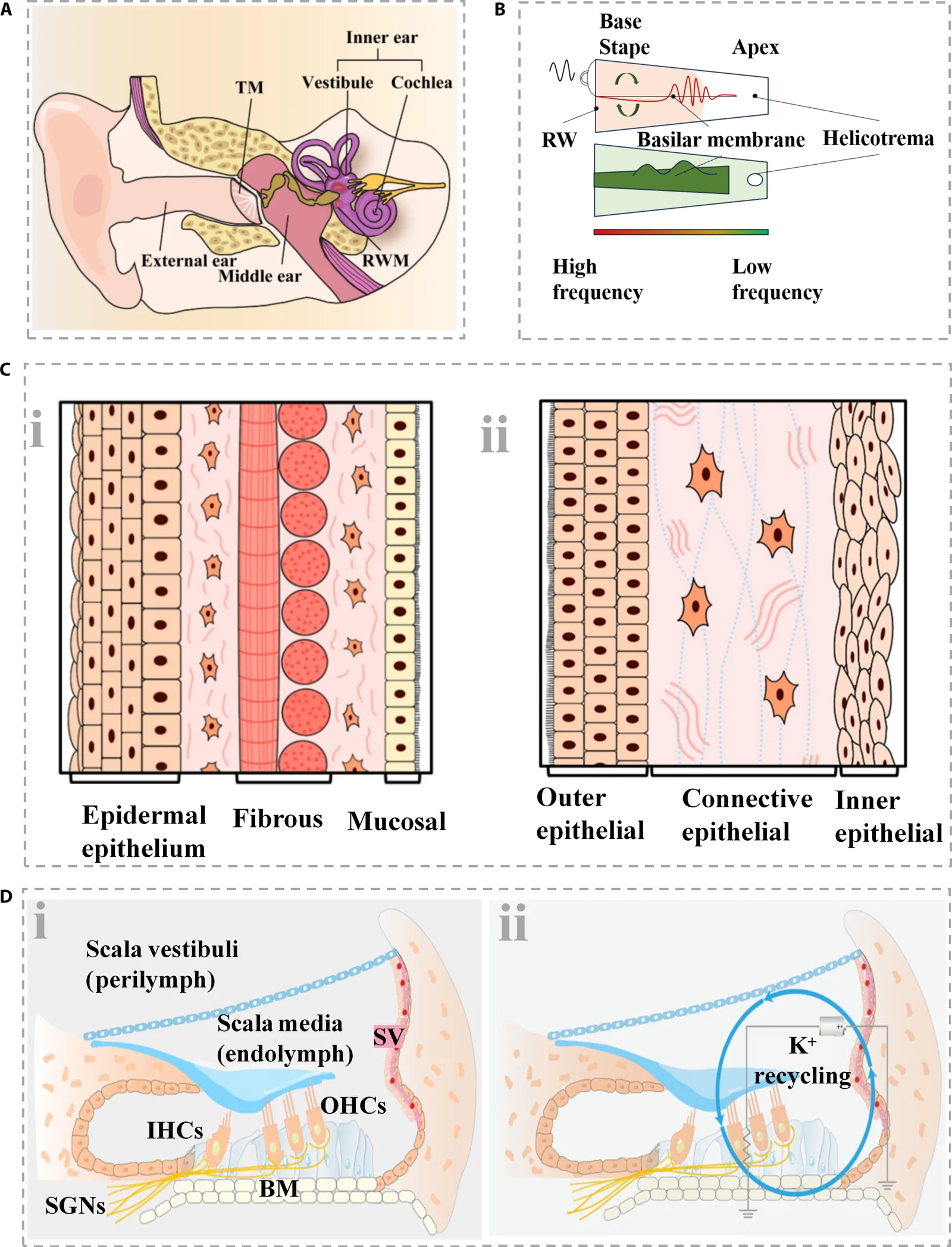
(A) Schematic of human ear anatomy [236]. Copyright 2025, The Authors. (B) Schematic of sound wave travel principle. Reproduced with permission [13]. Copyright 2022, The Authors. (C) (i and ii) Cross-sectional view of TM and RWM. Reproduced with permission [11]. Copyright 2023, The Authors. (D) (i) Cross-sectional view of the cochlea. Reproduced with permission [15]. Copyright 2025, Wiley-VCH. (ii) SV functions as cochlear battery. Reproduced with permission [15]. Copyright 2025, Wiley-VCH. TM, tympanic membrane; RW, round window; OW, oval window; SV, stria vascularis; IHCs, inner hair cells; OHCs, outer hair cells; SGNs, spiral ganglion neurons; BM, basilar membrane.

Anatomically, the ear consists of the external ear, the middle ear, and the inner ear (Fig. 2A). The external ear and the middle ear are separated by the TM, which is a thin, concave tissue. TM contains epidermal epithelium and lamina propria, which includes fibroblasts and mucosal epithelium (Fig. 2Ci) [11]. The middle ear includes the tympanic cavity, malleus, incus, and stapes. The malleus is connected to the incus, whose opposite end connects to the stapes stem, and the stapes footplate leans against the opening of the oval window (OW). The inner ear and the middle ear are separated by the round window (RW) and the OW. These openings serve as gateways from the middle ear to the cochlea [12]. The RW membrane (RWM) and the OW membrane (OWM) cover them, respectively. The RWM is a 3-layer membrane composed of an outer epithelial layer, a middle connective tissue layer, and an inner epithelial layer (Fig. 2Cii) [11]. When acoustic waves are transmitted, the stapes footplate vibrates the cochlear perilymph through the OWM. This pressure is subsequently transmitted to the RWM, which vibrates in the opposite direction to balance the pressure. These vibrations are then delivered to the cochlea. The cochlea is a system of coiled tubes: the scala vestibuli, the scala media, and the scala tympani. Perilymph fills the scala vestibuli and the scala tympani, while endolymph fills the scala media. The scala tympani and the scala media are separated by the hair cell (HC)-covered basilar membrane. High-frequency sounds vibrate the basal part of the basilar membrane, whereas low-frequency sounds vibrate the sensory HCs of the apical part (Fig. 2B). HCs serve as the receptor organs in response to the vibration of the basilar membrane (Fig. 2Di). HCs can be divided into inner HCs (IHCs) and outer HCs (OHCs). IHCs are mainly responsible for converting mechanical vibrations into neural signals for transmission, and OHCs can enhance auditory sensitivity through mechanical amplification [13].

In the event of sound vibration, the stereocilia on the HCs bend in the direction of the longest one. This phenomenon results in the opening of the K^+^ channels, leading to depolarization, which subsequently triggers the opening of the voltage-gated Ca^2+^ channels, enhancing depolarization, promoting glutamate release, and depolarizing the sensory nerve. Apparently, different K^+^ concentrations between HCs and endolymph are vital for maintaining their functions. The stria vascularis (SV) plays an important role in safeguarding a high-potassium, positive potential environment. It is a highly vascularized epithelial–mesodermal tissue located in the lateral wall of the cochlea. The SV is composed of marginal, intermediate, and basal cells. The Na-K-2Cl cotransporter 1 and the Na/K-ATPase (adenosine triphosphatase) pump are jointly involved in K^+^ transportation to the endolymph, facilitating the formation of the +80-mV endocochlear potential (EP) [14]. After being transported from the SV to the endolymph and entering HCs, K^+^ drains from HCs into the perilymph and returns to the SV through gap junctions to form the K^+^ cycle (Fig. 2Dii) [15]. Furthermore, the BLB, a physicochemical barrier between the body’s circulatory system and the cochlea, functions to maintain a stable inner ear environment [16]. In the BLB, the basement membrane surrounds the endothelium, and pericytes surround the basement membrane. The BLB presents similarities with the blood–brain barrier (BBB) in both structure and cell types. These barriers both have tight junctions composed of similar proteins connecting the endothelial cells [17].

We will briefly introduce several other structures of the inner ear that have the potential to serve as administration sites. The utricle is a receptor for linear accelerating and decelerating motion. It is a slightly compressed and elongated oval capsule connected to the saccule [18]. The endolymphatic sac is the terminal expansion segment of the membranous labyrinth and connects with the utricle for endolymph absorption and drainage. Endolymphatic sac dysfunction can lead to endolymph accumulation, a common phenomenon in Ménière’s disease [19]. Semicircular canals are 3 two-thirds circular tubes, with both ends opening to the vestibule. They are composed of bony semicircular canals and semicircular ducts. According to spatial location, there are lateral, superior, and posterior semicircular canals (PSCC). These canals monitor and regulate angular head acceleration [20].

### Pathological factors causing hearing loss

Conductive hearing loss is characterized by an interruption in transmitting sound waves to the cochlea, while SNHL is characterized by the onset of hearing impairment due to the malfunction of the structures responsible for translating neural impulses to the brain [1]. Multiple factors are related to the development of SNHL, such as noise exposure, aging, genetic susceptibility, drug ototoxicity, viral infections, hyperbilirubinemia, and Ménière’s disease.

In the field of SNHL, noise-induced hearing loss (NIHL) is an important subset characterized by hearing loss subsequent to noise exposure. NIHL can be caused by exposure to a potent acoustic stimulus or developing as a long-term process. Although moderate-intensity exposures cause only a temporary threshold shift, continuous damage may result in the loss of HCs and SG neurons (SGNs), which leads to a permanent threshold shift [21]. In addition, noise can impact SV-maintained inner ear homeostasis. After noise exposure, increased nicotinamide adenine dinucleotide phosphate (NADPH) oxidase (NOX) subunit and interleukin-6 (IL-6) levels promote reactive oxygen species (ROS) generation, further causing SV dysfunction [22]. Moreover, cochlear resident macrophages exhibit pro-inflammatory characteristics in NIHL promotion. Activated resident macrophages present morphological changes and increase in number, and their migration to the HCs area leads to OHC loss and ribbon synapse damage [23]. Even worse, inflammation and ROS are 2 processes that mutually facilitate each other.

In age-related hearing loss (ARHL), organ and tissue degeneration such as HC loss and SV and SGN degeneration accelerate ARHL development [24]. These age-related damages accumulate and increase oxidative stress, which causes DNA, lysosome, and mitochondria damage, elevates ROS levels, and further promotes the onset of ARHL [25]. At the molecule level, multiple signaling pathways are involved in ARHL processing, such as sirtuin1 (SIRT1), phosphoinositide 3-kinase (PI3K)/protein kinase B (Akt)/mammalian target of rapamycin (mTOR), nuclear factor erythroid 2-related factor 2 (Nrf2)/antioxidant response element (ARE), and atrial natriuretic peptide (ANP)/natriuretic peptide receptor A (NPRA)/cyclic guanosine monophosphate (cGMP)/protein kinase G (PKG) signaling pathways. SIRT1 is a nicotinamide adenosine dinucleotide (NAD)-dependent deacetylase; its activation protects HCs through autophagy restoration [26]. Low activity of Akt-mTORC1 hinders neonatal murine HC formation [27]. Connexin-26 gene gap junction protein β2 (*GJB2*) gene mutation affects the Nrf2/ARE signaling pathway, lowers Nrf2 or Nrf2/ARE-controlled heme oxygenase-1 (HO-1) expression, and accelerates ARHL progression [28]. Activation of the ANP/NPRA/cGMP/PKG signaling pathway protects SGNs and facilitates their regeneration [29].

Genetic factors are also important pathological determinants of SNHL [30]. Hereditary hearing loss can be classified into syndromic hearing loss and nonsyndromic hearing loss. Based on the mode of inheritance, there are autosomal recessive, autosomal dominant, X-linked, mitochondrial four patterns [31]. Among the various forms of hereditary hearing loss, several causative genes have been identified as promising therapeutic targets. One notable example is autosomal recessive deafness 9 (DFNB9), which is caused by mutations in *OTOF*. *OTOF* encodes otoferlin, a synaptic protein essential for maintaining normal IHC function [2]. Encouragingly, *OTOF* gene therapy has not only been shown to effectively alleviate hearing loss in animal models but also demonstrated therapeutic efficacy in pediatric patients [32,33]. Adeno-associated virus (AAV)-*OTOF* injection restored a 5-year-old patient hearing and recognizing speech [32]. In addition to *OTOF*, several other genes have been frequently implicated in hereditary hearing loss. *GJB2*, solute carrier family 26, member 4 (*SLC26A4*), and usherin (*USH2A*) are the commonly affected genes. The deletion of G at positions 30 to 35 (mutation 35delG) of the *GJB2* gene was commonly detected in *DFNB1* families; this mutation leads to the complete loss of connexin 26 (Cx26) function and further causes cochlear developmental disorders [34–37]. *SLC26A4* encodes pendrin, which is essential for maintaining inner ear homeostasis, and mutations in this gene may result in autosomal recessive deafness 4 (DFNB4) [38]. *USH2A* encodes usherin; the point deletion in exon 13 (c.2299delG) is a common pathogenic variation of *USH2A* [39]. Human frameshift mutation c.2299delG is basically equal to c.2290delG mutation in mouse model. Studies have shown that the c.2290delG mutation leads to mislocalization of truncated protein in the HCs and disorganization of stereocilia bundles [40].

Drug ototoxicity is considered another crucial pathological factor. Aminoglycoside antibiotics, such as gentamicin and neomycin, can induce HC death and impair hearing. Aminoglycoside antibiotics enter the endolymph with the help of their main transporter, megalin, and reach HCs through the mechano-electrical transduction (MET) channel [41]. The damaging mechanism of aminoglycoside antibiotics is thought to be related to the calcium uptake by mitochondria and lipid metabolism disorders [42,43]. Platinum-based drugs, for example, cisplatin, can enter the inner ear with the assistance of copper transporter 1 and organic cation transporter 2. Thereafter, cisplatin enters the HCs, promoting the release of inflammatory factors like tumor necrosis factor-α (TNF-α), IL-1β, and IL-6. These factors collectively break the balance between free radicals and antioxidant systems, facilitate ROS generation, and further damage HCs through cytoplasmic organelle damage and DNA damage [44].

In addition to ototoxic drugs, viral infection is another promoter of hearing loss. Cytomegalovirus (CMV) is one of the most common nongenetic factors contributing to SNHL. CMV infection results in up-regulated pro-inflammatory factor expression and SGN loss, and finally leads to SNHL [45]. Furthermore, SNHL is part of a group of clinical signs of congenital rubella syndrome, which is caused by the rubella virus. Rubella virus may cause SNHL by changing the expression of sensory development-related genes. For instance, myosin VIIA (*MYO7A*) was observed to decline in both primary endothelial cells of fetuses and adults after rubella virus infection [46].

Hyperbilirubinemia is also a risk factor that needs to be considered. Unconjugated bilirubin causes auditory nerve fiber demyelination and SGN density decline, as well as necrosis and pyroptosis in a rat injury model [47]. Ménière’s disease requires attention, as patients exhibit varying degrees of endolymphatic hydrops. Neuronal toxicity is considered the main mechanism of Ménière’s disease, as histopathological analysis revealed severe SG injury and loss [48]. The molecular mechanisms can be attributed to nitric oxide synthase-triggered apoptosis through caspase-dependent and caspase-independent pathways, activation of ROS, and endolymphatic hydrops-mediated stress [49].

## Drug Delivery Routes

**Fig. 3.**
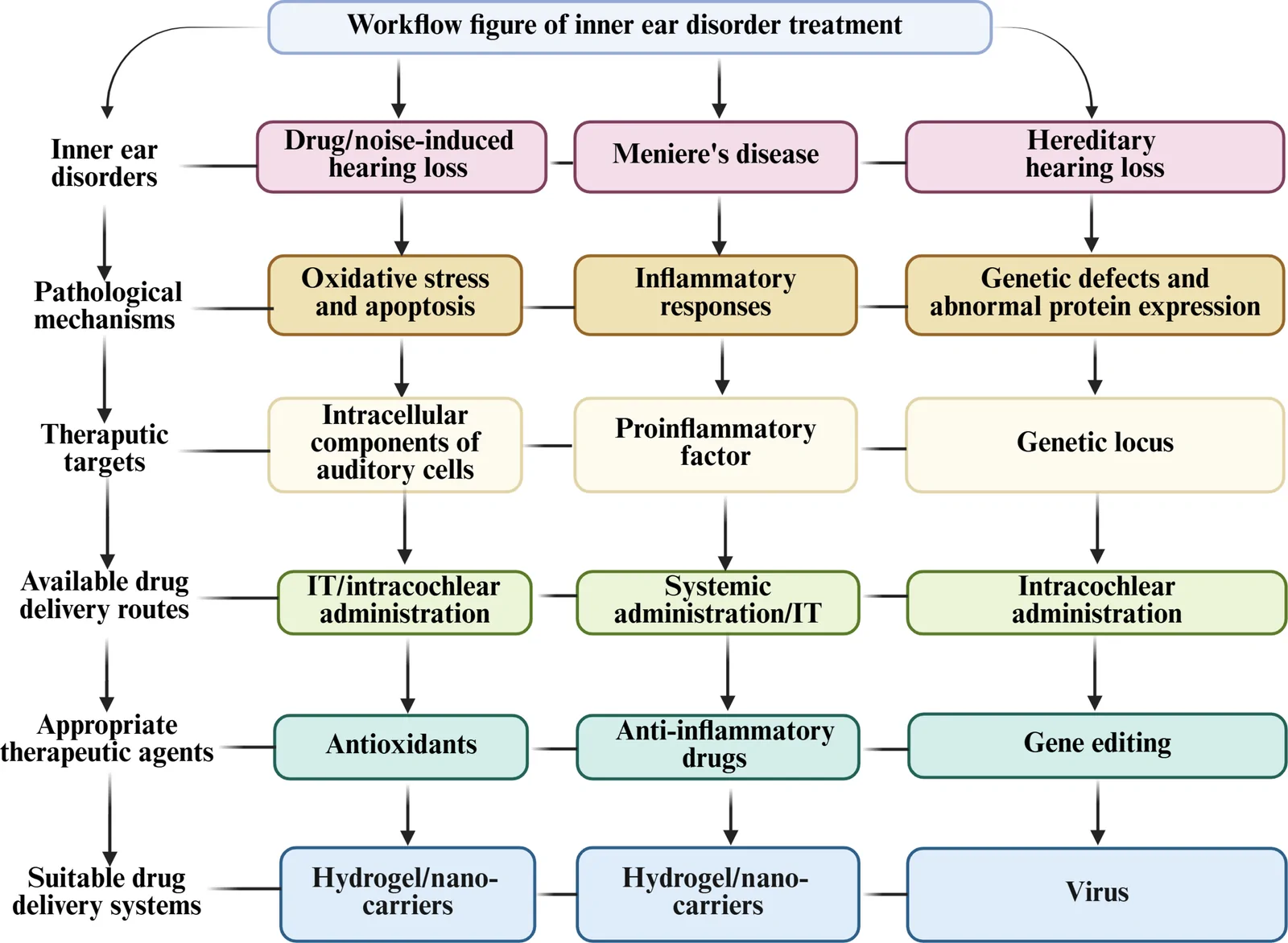
Workflow for selecting delivery routes, carrier types, and therapeutic agents based on disease types and therapeutic targets. (Figure was created with BioRender.com.)

The selection of an appropriate drug delivery route to the inner ear is intricately linked to the pathophysiology of specific ear diseases. In cases of drug- or noise-induced hearing loss, antioxidant-loaded nanocarriers and injectable hydrogels are likely to be employed to alleviate ROS-associated inner ear damage through intratympanic injection (IT) or intracochlear administration. For patients with Ménière’s disease who suffer from inflammation-related damage [50], both systemic administration and IT delivery are considered appropriate routes. Regarding hereditary hearing loss, a more precise intracochlear administration route, such as PSCC injection, is required for effective restoration of auditory function (Fig. 3).

**Fig. 4.**
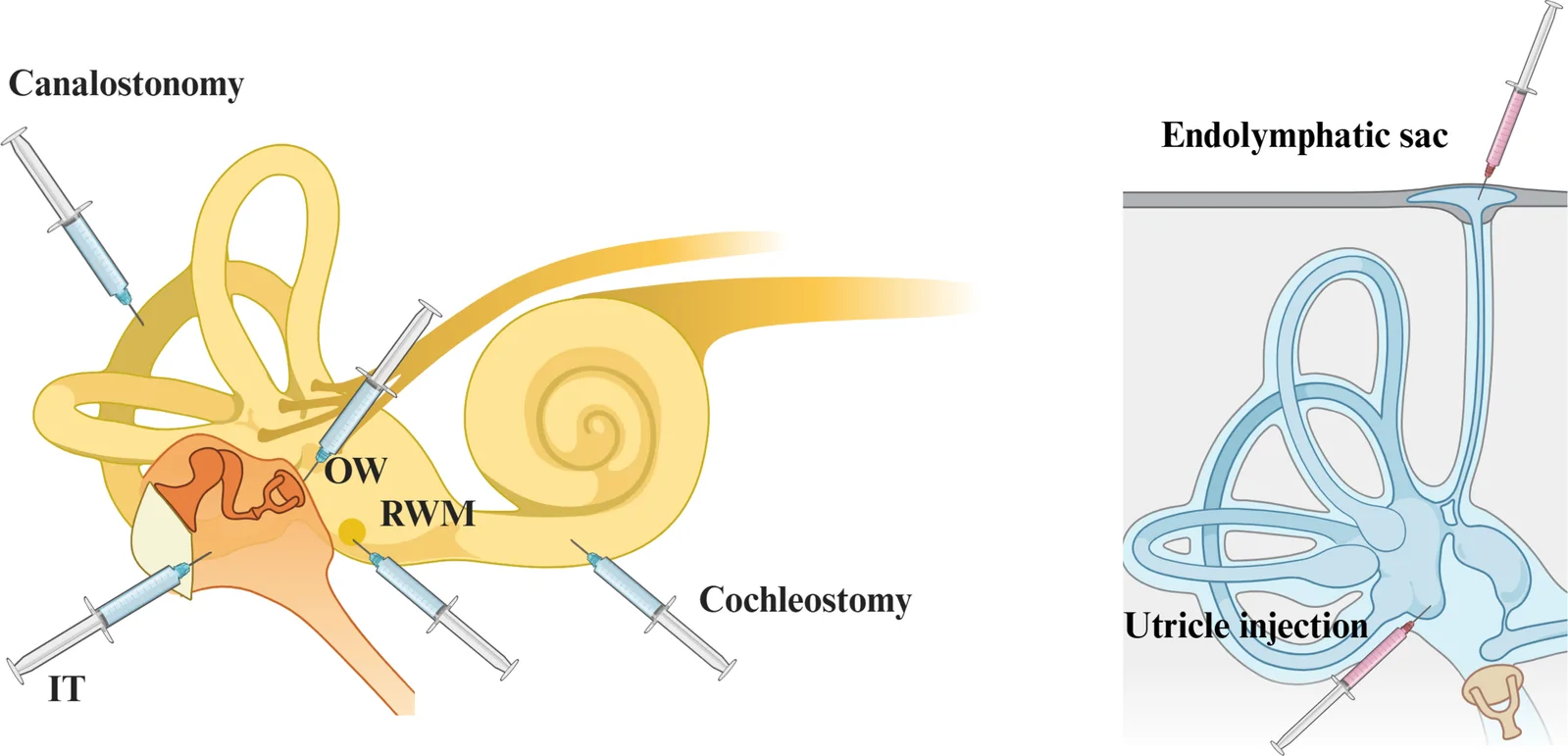
Schematic representation of diverse delivery routes for inner ear treatment. IT, intratympanic injection; OW, oval window; RWM, round window membrane. (Figures were created with BioRender.com.)

Diverse inner ear drug delivery routes have been studied to achieve better therapeutic effects. These routes are primarily divided into systemic drug delivery, drug delivery through the TM, intracochlear drug delivery (Fig. 4), and CI. We will next describe these administration routes in more detail.

### Systemic drug delivery

Systemic drug delivery includes multiple methods such as intravenous, intramuscular injection, and oral routes [10]. However, the complicated structure of the BLB impedes the delivery of therapeutic agents. Therefore, some patients require more precise treatment.

### Drug delivery through TM

To reduce the adverse effects engendered by repeated systemic administration, more precise drug delivery routes—specifically delivery through the TM—have been studied. In detail, drugs can cross the TM through noninvasive or invasive ways [51]. Through the noninvasive method, drugs need to cross the TM and reach the middle ear, then permeate through the RWM or OWM, and eventually reach the inner ear. However, it is a challenge for the therapeutic agent to cross the epidermal epithelium-based TM outermost layer [11]. Hence, an invasive method, IT, is required, which can deliver drugs/drug delivery systems directly to the middle ear or place them at the RWM. Zou et al. [52] used silver nanoparticles (NPs) to observe the efficiency of IT. After IT, silver NPs crossed the OW and RW, presenting a gradient concentration from the middle ear to the inner ear. Compared with systemic administration, dexamethasone (DEX) labeled with a fluorescent marker exhibited moderate fluorescence 12 h post-injection [53]. As a convenient and effective administration route, IT is preferred in both research and clinical areas. Furthermore, a combination of systemic administration and IT enables rapid detection of mimic drug fluorescent signal, which is approximately 3 min post-administration [54]. Nevertheless, IT still carries risks of pain, tongue numbness, and vertigo. To reduce these complications, it is preferred to perform IT at the anterior–inferior quadrant of the TM [55]. Additionally, for children who need multiple treatments, a tympanostomy tube is a good choice to alleviate discomfort. Moreover, the operator’s experience is of great importance to avoid inaccurate injection.

### Intracochlear drug delivery and CI

To further elevate inner ear drug concentration, intracochlear drug delivery has attracted much attention. After intracochlear drug delivery, drug concentration in the perilymph was found to be 7 times higher than that after IT [56]. Intracochlear drug delivery could be accomplished through various approaches. Cochleostomy is viable at the basal turn of the cochlea, and trans-OWM is a considerable choice, but related reports are limited [57]. Trans-RWM is more common, directly placing/injecting drugs on/through the RWM. Drug delivery systems were still detected 8 weeks after administration through RWM incision [58]. In addition, the mastoid approach offers a novel idea for trans-RWM drug delivery through the retroauricular area. Compared with IT and intraperitoneal drug delivery, the mastoid approach presents the best therapeutic effect, achieving a maximum DEX concentration in inner ear perilymph of 92.53 ± 4.68 μg/l [59]. Generally, these administration methods have the advantages of precise drug delivery and reducing side effects. However, variations in size and structure among individuals complicate the location of the RWM. The intricate and delicate nature of the inner ear structure necessitates that operators have extensive experience. Drug clearance via the eustachian tube becomes an issue for reaching effective therapeutic concentrations as well. Furthermore, a decreasing trend of dye distribution from base to apex after RWM injection indicates uneven drug concentration in the cochlea [60]. Hence, Wang et al. [61] modified the poly(lactic-co-glycolic acid) (PLGA) NPs by targeting peptide A665, enabling the NPs to bind a protein exclusively expressed by OHCs. This modification contributes to drug redistribution and preserves OHCs from all turns of the cochlea. In gene therapy, uneven drug distribution could potentially result in constrained viral transduction. Therefore, utricle injection has been studied. The saccular anatomy of the utricle allows easier access to the endolymphatic compartment, which exhibits a slightly higher transduction rate than RWM injection while minimizing the risk of damaging cochlear structures [62]. Nevertheless, reports based on this administration method are relatively scarce, possibly because this method has limitations regarding operations.

Canalostomy refers to surgical pore creation toward the vestibular semicircular canal, mostly at the PSCC. Injection through the PSCC is a perilymph selectively targeted delivery route. In vivo real-time monitoring showed that gold NPs injected via the PSCC primarily distributed in the scala vestibuli and scala tympani rather than the scala media, which may facilitate perilymph-targeted administration [63].

The endolymphatic sac is another potential administration site exposed through the extradural posterior cranial fossa approach. After exposing the operculum aperture (where the endolymphatic sac extends toward the sigmoid sinus), a small incision is made for drug delivery. The injected fluorescent indicators were primarily distributed in the vestibule instead of the cochlea, offering a therapeutic possibility for vestibular lesion-related diseases [64]. Cochleostomy could deliver the agent to the scala tympani and/or the scala media endolymph. Kim and Ricci [65] reported a chemo-mechanical cochleostomy, which can preserve hearing for the in vivo functional imaging.

CI is a crucial treatment for SNHL, which is commonly combined with cochleostomy. During the CI procedure, part of the mastoid is removed, and a tiny hole allows electrode installation [66]. However, as an invasive procedure, CI may lead to complications such as injury, foreign body response, and cochlear fibrosis. To mitigate these effects, multiple strategies have been developed, including perioperative pharmacological interventions, electrode-array design improvements, and local drug delivery approaches. Preoperative systemic glucocorticoids and antibiotics are supposed to ameliorate immune responses and bacterial infections [67]. IT administration of triamcinolone-acetonide suspension, 40 mg/ml, has also been shown to protect against cochlear fibrosis [68]. To minimize insertion-related trauma, appropriate implant insertion depth is critical. Therefore, Mertens et al. [69] developed a diameter and width-based elliptic circular approximation system to accurately predict a suitable insertion depth. Besides, a forward-facing optical coherence tomography (OCT) probe was studied to provide the real-time image of the cochlear lumen, thereby enabling timely adjustments and reducing unnecessary tissue damage [70]. Beyond surgical guidance, optimization of electrode-array design represents another crucial strategy for reducing insertion trauma. Electrode diameter around 0.4 to 0.5 mm, with 20 electrodes on a single insertion length (25 mm), is recommended to minimize the insertion injury [71]. Itawi et al. [72] developed a steerable thin-film electrode array with gold electrodes. The large surface area of gold electrodes, adjustable stiffness, and curvature made this 10-μm-thick electrode a promising option for future CI applications. In addition to mechanical optimization, local drug delivery systems have emerged as an attractive strategy for suppressing fibrosis. From the perspective of drug delivery system design, electrode coating, drug-loaded electrodes, and connecting them with drug reservoirs or pump systems are 3 feasible options to alleviate fibrosis [73]. Among these 3 choices, electrode coating has aroused great attention. The coatings are intended to reduce fibrosis by decreasing friction and minimizing the contact between inner ear tissue and the implants, as Jensen et al. [74] found that cells are more likely to adhere and proliferate on the surface of platinum electrodes, rather than the poly(dimethylsiloxane) (PDMS) coating. To date, diverse polymers with good biocompatibility and low cytotoxicity like hydrogels [75] are used in electrode coating. Horne et al. [76] reported a hydrogel coating, in which poly(carboxybetaine methacrylate) was simultaneously photo-grafted and photo-polymerized onto PDMS. This coating effectively reduced the capsule thickness during 1 year of the implantation with its anti-fouling property. Furthermore, therapeutic agents could be loaded in the electrode coating to further alleviate fibrosis. Ren et al. [77] prepared a porous methacrylated PDMS (MA-PDMS)-coated cochlear electrode filled with gelatin methacryloyl (GelMA), Ti_3_C_2_T_x_ MXene (MXene), and therapeutic agents, which effectively improved cell condition and viability. Despite these advances in anti-fibrotic strategies, real-time monitoring of fibrosis progression following CI remains challenging, while de Rijk et al. [78] pointed out that complex impedance is becoming a novel marker for real-time tracking. Their findings suggest that impedance-based monitoring may provide a noninvasive and clinically translatable approach for assessing cochlear fibrosis after implantation.

## Therapeutic Drugs Used in Inner Ear Disorders

While the route of administration determines how therapeutic agents gain access to the inner ear, the choice of drug indicates the biological targets and therapeutic outcomes of treatment. Anti-inflammatory drugs, antioxidants, nanozymes, and factors have attracted considerable attention. We will next describe them in detail.

### Anti-inflammatory drugs

**Fig. 5.**
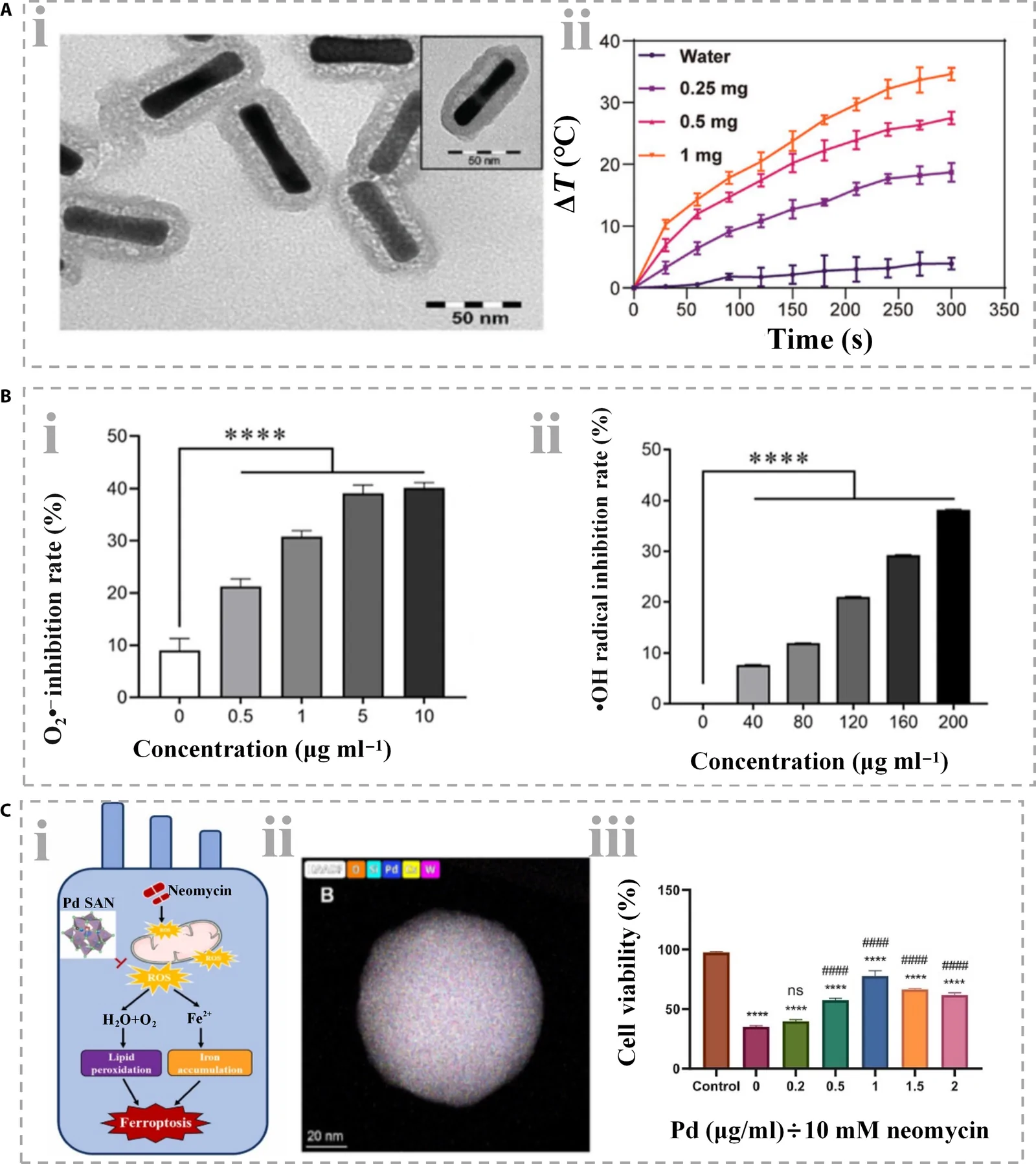
(A) (i) Transmission electron microscopy (TEM) images of DEX-loaded nanocomposite [83]. Copyright 2024, The Authors. (ii) Nanocomposite photothermal effect characterization [83]. Copyright 2024, The Authors. (B) (i) Preparation procedure of the AMNPs [108]. Copyright 2024, American Chemical Society. (ii and iii) AMNP inhibition effects of O_2_•^–^, •OH radicals. Reproduced with permission [108]. Copyright 2024, American Chemical Society. (C) (i) Protective effect of the self-assembly Pd SAN against neomycin-induced damage. Reproduced with permission [112]. Copyright 2024, Elsevier. (ii) TEM image of the Pd SAN [112]. Copyright 2024, Elsevier. (iii) Pd SAN protected HEI-OC1 cells from neomycin-induced damage. Reproduced with permission [112]. Copyright 2024, Elsevier. TEM, transmission electron microscope; DEX, dexamethasone; DI water, deionized water; 1,8-DHN, 1,8-dihydroxynaphthalene; NaIO_4_, oxidizing agent; AMNPs, allomelanin nanoparticles; Pd SAN, palladium single-atom nanozyme; HEI-OC1 cells, house ear institute-organ of Corti 1 cells.

Glucocorticoids, exerting their effects through interactions with glucocorticoid receptors, play an important role in SNHL treatment. Glucocorticoid receptors exist in the inner ear of both normal individuals and NIHL patients, offering a possibility for glucocorticoid therapy [79]. DEX is one of the most widely used glucocorticoids in inner ear therapy, exhibiting remarkable anti-inflammatory properties. It not only lowers the levels of inflammatory factors but also exhibits anti-apoptotic effects. When the concentration is higher than 5 μg/ml, DEX can preserve both IHCs and OHCs against 0.3 M gentamicin damage. This protective effect is mediated through the down-regulation of the apoptosis activator B cell lymphoma-2 (Bcl-2)-associated X protein (Bax) [80]. Nevertheless, the low water solubility of DEX promotes its conjugation with liquid crystalline NPs to accelerate DEX delivery [81]. In addition, Yang et al. [82] loaded DEX into phospholipid-based cationic-polyethyleneglycol (PEG) NPs, which yielded superior suppression of inflammatory factors. Furthermore, Mustafa et al. [83] prepared saponin-coated nanocomposite-gold nanorods enclosed in mesoporous silica for DEX loading (NPs/DEX) (Fig. 5Ai). Gold nanorods possess photothermal conversion functions [84,85], thereby facilitating drug release and RWM permeation (Fig. 5Aii).

### Antioxidants

Sodium thiosulfate [Na_2_S_2_O_3_ (STS)] is a cisplatin-neutralizing agent that can effectively inactivate cisplatin by binding it [86]. Patients receiving STS treatment exhibited a significantly lower risk of suffering from ototoxic effects during platinum-based chemotherapy [87].

Glutathione [γ-l-glutamyl-l-cysteinyl-glycine (GSH)] is a crucial reducing agent involved in various biological processes, including antioxidant activity, anti-inflammatory activity, and DNA synthesis [88]. Kim et al. [89] found that bucillamine facilitates GSH synthesis by inducing the intranuclear translocation of Nrf2, which subsequently increases γ-glutamylcysteine synthetase and GSH synthetase expression, thereby elevating the level of intracellular GSH. *N*-acetylcysteine (NAC) serves as an indirect GSH supplement [90]. NAC can attenuate ROS level elevation in HCs’ mitochondria. It has been reported that 20 mM NAC could alleviate 50 mM cisplatin-induced ototoxicity [91].

As a classical analog of glutathione peroxidase (GPx), ebselen presents excellent ROS- scavenging and anti-inflammatory properties. A phase 2 trial (NCT01444846) showed that 400 mg of ebselen, twice a day, could alleviate noise-induced temporary threshold shifts at 4 kHz [92]. In another delivery system, ebselen liposomes were loaded in GelMA microspheres coupled with a polydopamine (PDA) layer. This delivery system exhibited a 93 ± 2% survival rate of OHCs at 32-kHz frequency [93]. Additionally, a novel class of isoselenazolones with GPx-like characteristics has been shown to protect HCs by suppressing plasma pro-inflammatory factors (malonaldehyde, IL-6, and TNF-α) levels while alleviating superoxide dismutase (SOD) activity reduction [94].

α-Lipoic acid (ALA), also known as thioctic acid (C_8_H_14_O_2_S_2_), facilitates an increase in intracellular GSH levels and GPx expression. This short-chain fatty acid exhibits ROS scavenging, antioxidant protein level adjustment, and anti-inflammatory properties [95]. In a cisplatin-induced hearing loss model, a PDA-based nanogel (PDA@microcarriers-ALA) evidenced the inner ear protective effect of ALA. 2′,7′-Dichlorofluorescein diacetate (DCFH-DA) detection revealed that the PDA@microcarriers-ALA pretreated group showed a similar fluorescent signal to that of the control group, while cisplatin-treated house ear institute–organ of Corti 1 (HEI-OC1) cells exhibited stronger fluorescence, indicating a higher level of ROS [96]. Apart from this, ALA’s apoptosis suppression effect alleviates mitochondria ROS level elevation. The up-regulation of Bcl-2 and down-regulation of Bax together prevent apoptosis [97]. Jung et al. [98] also found that antioxidant proteins, including HO-1, SOD-1, and SOD-2 levels, all exhibited an increasing tendency after ALA-NP administration.

Vitamin C and vitamin E are crucial water- and lipid-soluble vitamins, respectively. Vitamin C possesses both anti-inflammatory and antioxidant activities; it is hypothesized to reduce ROS generation by inhibiting the activation of ROS/nuclear factor-κB (NF-κB) pathway [99]. Long-term oral vitamin C administration (150 mg/kg/d) protects hearing by inhibiting NOD-like receptor family pyrin domain containing 3 (NLRP3) inflammasome activation [100]. Rats receiving vitamins A, C, and E and Mg^2+^ showed significant recovery after noise exposure for 30 d. The antioxidant effects of the vitamins were evidenced by reduced levels of pro-apoptotic caspase-3 and Bax, as well as elevated levels of GPx1 and Bcl-2 [101].

Astaxanthin is a natural marine carotenoid that exhibits anti-inflammatory and antioxidant properties. Astaxanthin possesses 100 to 500 times more potent oxygen radical absorbance capacity and 10 times higher free radical inhibitory activity than α-tocopherol [102]. In hearing loss treatment, astaxanthin mainly combats cisplatin-induced ototoxicity by activating the Nrf2-mediated pathway. Furthermore, it can down-regulate apoptotic-related gene expression [103]. Therefore, it is a promising agent. However, crossing the RWM remains a challenge due to its hydrophobicity and instability. Gu et al. [104] attempted to solve this problem by loading it into poly(propylene sulfide)-PEG-based NPs, which could effectively deplete ROS.

Various plant-derived drugs exhibit outstanding antioxidant effects as well. Chen and colleagues [105] loaded panax, saponins, and tanshinone IIA into space-station-like composite NPs (CCC@mPP NPs), which are capable of preserving HCs against aminoglycoside antibiotics or cisplatin-induced damage. As a kind of Chinese herbal medicine, berberine can be used for hearing loss treatment. Zhao et al. [106] prepared a ROS-responsive NP for berberine inner ear administration against NIHL. It was found that 0.01 mg/ml NPs could reduce ROS levels by 50.73%. In addition, decreasing levels of Berberine chloride has the properties of apoptosis suppression, mitochondrial membrane potential maintenance, and reduction of ROS accumulation. Pretreatment with 0.1 or 1 μM berberine chloride could prevent aminoglycoside antibiotic-induced OHC loss and stereocilia degeneration [107].

Allomelanin is a nitrogen-free melanin that usually exists in fungi. It can be artificially synthesized from 1,8-dihydroxynaphthalene (1,8-DHN). Chen et al. [108] prepared 1,8-DHN-based allomelanin NPs (AMNPs) (Fig. 5Bi), which exhibited outstanding free radical scavenging effects (Fig. 5Bii and iii). AMNPs present protective effects against both cisplatin-induced damage and NIHL. After treatment with AMNPs, HCs from the apex and middle regions were significantly preserved. Moreover, AMNPs help in alleviating SGN loss and improving IHC survival rate in NIHL, which might be attributed to the regulation of the *Stat1* gene and the Janus kinase (JAK)–signal transducer and activator of transcription (STAT) signaling pathway.

Nanozymes, a class of nanomaterials with enzyme-like activities, have wide applications in biomedical and therapeutic fields [109–111]. Huo et al. [112] prepared a palladium (Pd)-based single-atom nanozyme (SAN) exhibiting SOD and catalase (CAT) activities (Fig. 5Ci and ii). This nanozyme reduced apoptosis and maintained mitochondrial potential by alleviating neomycin-induced mitochondrial ROS level increases. It was demonstrated that 1 μg/ml of Pd performs the best protective effect against neomycin-induced damage (Fig. 5Ciii). In addition, Ge et al. [113] synthesized NAC-derived carbonized polymer dots (NAC CPDs) loaded within manganese porphyrin-doped metal–organic frameworks and then encapsulated with GelMA and PDA. This multifunctional nanozyme microcapsule exhibited SOD and CAT activities as well. It decreased mitochondrial depolarization, lowered ROS levels, reduced inflammation, and restored DNA damage.

### Factors

Brain-derived neurotrophic factor (BDNF), a neurotrophic protein, promotes neuron survival. Pisani et al. [114] pointed out the protective effects of human recombinant BDNF (rhBDNF) against cisplatin-induced damage by preserving paired ribbon synapses and OHCs. This protection is thought to be associated with the balance between tyrosine kinase B (TrkB) and p75 (another BDNF receptor whose activation promotes apoptosis). Increasing TrkB phosphorylation and decreasing p75 levels collectively indicate the antioxidant effects of rhBDNF. Neurotrophin-3 (NT-3) is a neurite-promoting factor. Both NT-3 and its receptor TrkC are crucial for maintaining the function of basal turn SG cells function [115]. Wang et al. [116] prepared a peptide-conjugated, PEG-based injectable hydrogel system for sustained NT-3 release, offering a new option for NT-3 inner ear delivery. Furthermore, Zhao et al. [117] conjugated pigment epithelium-derived factor (PEDF) to a prestin-targeting peptide 2 with N-succinimidyl-3-maleimidopropionate (PrTP2-SMP/PEDF), enabling its aggregation and sustained release around the OHCs, effectively reducing the loss of OHCs and SGNs.

## Drug Delivery Systems

**Fig. 6.**
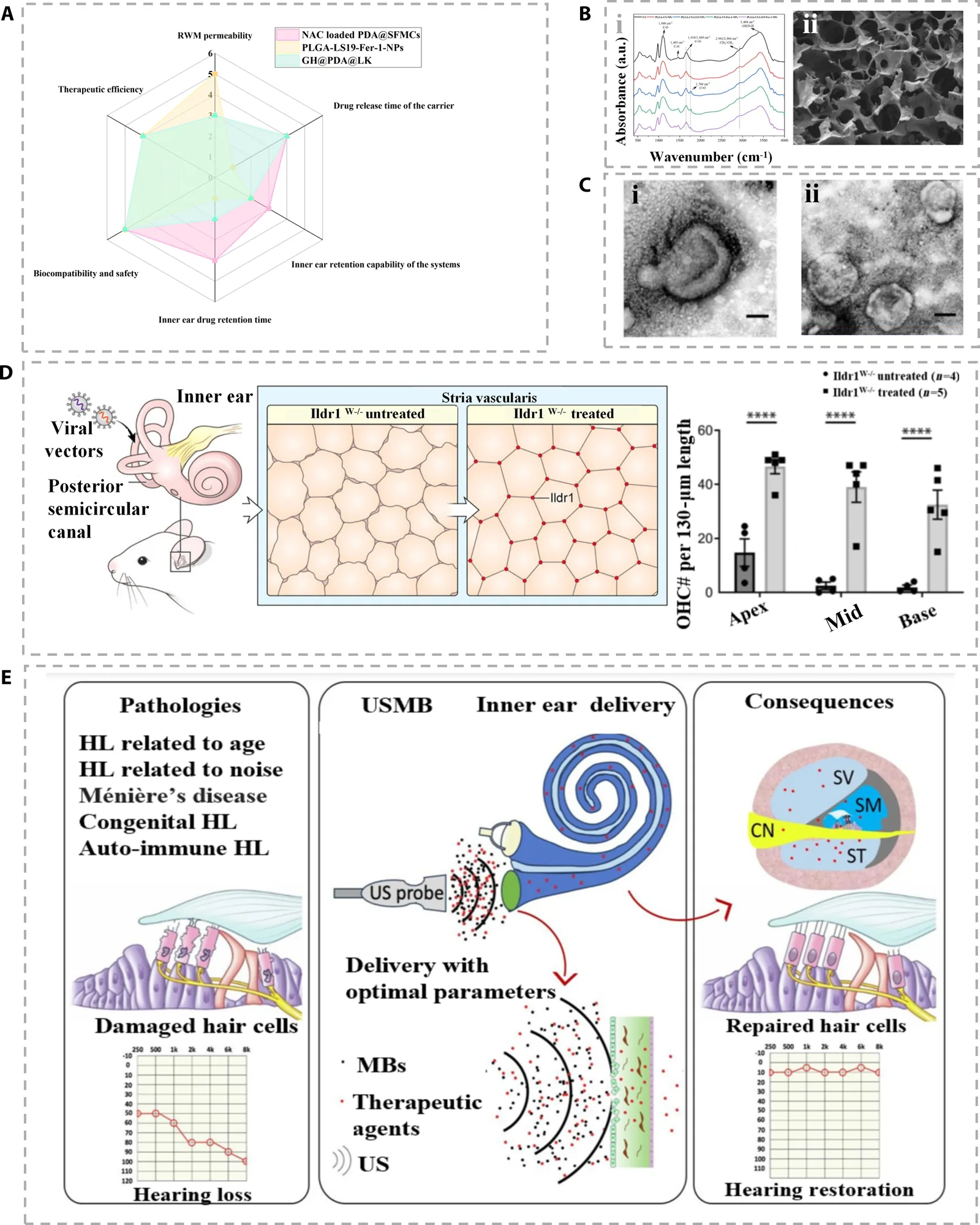
(A) Comprehensive evaluation of diverse inner ear drug delivery systems, visualized using a radar chart. Higher scores indicate better system performance [131,209,237]. (B) (i) Fourier transform infrared spectroscopy analysis of four groups of PLGA-CS gels. (ii) SEM images of PLGA-CS-LS19-Fer-1-NPs. Reproduced with permission [131]. Copyright 2023, Elsevier. (C) TEM images of mesenchymal stem cell-derived exosomes. Scale bars, 100 nm (i) and 50 nm (ii). Reproduced with permission [142]. Copyright 2024, The Authors. (D) AAV-mediated gene replacement therapy improved auditory function in a mouse model. Reproduced with permission [149]. Copyright 2023, The Authors. (E) Schematic of USMB strategy for inner ear drug delivery. Reproduced with permission [155]. Copyright 2023, Elsevier. RWM, round window membrane; NAC, *N*-acetylcysteine; PDA@SFMCs, silk fibroin-polydopamine (PDA)-composited inverse opal microcarriers; PLGA-LS19-Fer-1-NPs, ferrostatin-1-loaded chitosan poly(lactic-co-glycolic acid)-based, polypeptide LS19-coupled thermosensitive nanodelivery system; GH@PDA@LK, polydopamine layer-conjugated gelatin microsphere functionalized with both the nitric oxide precursor l-arginine and calmodulin-dependent kinase II inhibitor KN93; SEM, scanning electron microscope; TEM, transmission electron microscope; AAV, adeno-associated virus; IHC, inner hair cell; OHC, outer hair cell; ABR, auditory brainstem response; *Ildr1^w−/−^*, a model of human DFNB42, nonsyndromic autosomal recessive hereditary hearing loss associated with *ILDR1* variants; US, ultrasound; MBs, microbubbles; USMB, ultrasound-meditated microbubble.

Although the therapeutic agents are the primary determinants of treatment efficacy, successful inner ear therapy also depends on the ability to deliver and maintain the drugs at the target site in a controlled and sustained manner. Consequently, increasing attention has been directed toward the development of advanced carrier systems capable of improving drug stability, prolonging residence time, and enhancing intracochlear distribution. A variety of drug delivery systems, including nanocarriers, injectable hydrogels, and micropumps, are under investigation. We summarize these approaches for inner ear delivery (Table 1) and assess several drug delivery systems (Fig. 6A) in order to provide a comprehensive overview.

**Table 1.** Comparative efficacy of diverse inner ear drug delivery systems

Carriers	Materials	Administration route	Dosage	Biocompatibility	Drug retention time	Object	In vivo efficacy	Ref.
Nanocarrier	UC-MSC-derived Exos (30–150 nm)	Trans-RWM	20 μg in 10 μl PBS	In vitro, 30 μg/ml Exos significantly protected the cochlear sample from the middle turn against the damages of 0.5 mM neomycin for 24 h	Not mentioned	Wild-type C57BL/6 mice, 7-d-old, preinjected subcutaneously with neomycin (dissolve in sterile saline) once per day for 5 consecutive days at a dose of 200 mg/kg Intraperitoneal injection of 3-MA at a dose of 30 mg/kg, administrated 3 times at 24 h before, 2 h before and immediately after administration	(1) Exos significantly reduced the hearing threshold at 4, 8, 16, 24, and 32 kHz (2) Exos-alone-treated mice showed no hair cell loss in all turns of cochlear tissue	[142]
Nanocarrier	SS-31 modified liposomes (149.4 ± 3.1 nm) loaded with MC (EE: 93.4 ± 2%; LE: 8.5% ± 0.3%)	Treated in embryo medium	SS31-Lp and 5 μM MC pretreated for 1 h	No significant damage to HCs was found upon chronic SS31-Lp at concentrations ranging from 0.1 mg/ml to 1.0 mg/ml	Not mentioned	Zebrafish, 5-d post-fertilization, treated with 200 or 2 μM of GT for acute (1 h) or chronic exposure (6 h) after administration	(1) Pre-treatment with SS31-Lp/MC significantly increased the number of surviving hair cells from 29.3% (GT group) to 71.0% (SS31-Lp/MC group) (2) The survival rate of HCs increased remarkably from 55.6% (GT group) to 77.7% (SS31-Lp/MC group).	[147]
Viral vector	AAV2.7m8-Cmv-Ildr1-EGFP AAV8BP2-Cmv-Ildr1-EGFP	PSCC injection	0.5 μl AAV2.7m8-Cmv-Ildr1-EGFP (2.91 × 10^9^ GCs) 0.5 μl AAV8BP2-Cmv-Ildr1-EGFP (0.58 × 10^9^ GCs)	GFP expression was detected in IHCs and OHCs and supporting cells in the organ of Corti after AAV2.7m8-GFPand AAV8BP2-GFP were injected in wild type mice.	ILDR1 expression was detected in the organ of Corti and marginal cells of the stria vascularis 2 months post-treatment and appears to be long lasting	Neonatal *Ildr1*^w−/−^ mice	(1) A statistically significant difference of ABR test at 8 kHz between treated and untreated animals 2 weeks post-treatment (2) A statistically significant difference of ABR test between the treated and untreated animals at 8 kHz (94.0 ± 2.08 dB SPL vs. 100 ± 0.00 dB SPL, respectively, 2 months post-treatment	[149]
Nanocarrier and hydrogel	Fer-1-loaded PLGA-LS19-NPs (173.53 ± 10.20 nm) dispersed into the CS-GP hydrogel (Fer-1 EE: 71.1% at average, DL: 5.38% at average)	IT (3 d before noise exposure)	5 μl	(1) CCK-8 tests demonstrated that the 4 groups of gels did not reduce the cell viability of HEI-OC1 cells at 24 and 48 h (2) Calcein/PI staining showed that HEI-OC1 cells grew well, and scarcely any PI-positive cells were observed	NP residence for at least 1 d.	Female C57BL/6J mice, 8-week-old, weighed 17 to 20 g, treated with 2–20 kHz, 105 ± 1 dB, 2 h	(1) The mean ABR threshold of the groups loaded with Fer-1 was decreased by approximately 12 dB (2) The Fer-1-loaded thermosensitive nano delivery system significantly alleviated OHC loss compared with the Fer-1-free thermosensitive nano delivery system at the base and hook turn of auditory epithelium	[131]
Injectable hydrogel	HGC-DEX thermogel formulations, contain 5 mg/ml of DEX	IT	100 μl	No evidence of inflammatory response was observed in the mucosa after IT of the HGC thermogels. Whole mount staining of IHCs and OHCs did not show cellular loss or death in any turns of the cochlea	About 1 d	Male albino guinea pigs, weighed 200–250 g	(1) HGC-DEX significantly increased the drug uptake to the cochlear tissue at 30 and 90 min of treatment (2) The thermogel formulations exhibited versatile release kinetics according to the drug type and concentration and could sustain drug release for up to 2 weeks	[182]
Multifunctional composite coating	MA-PDMS-coated cochlear electrode loaded with GelMA hydrogels containing MXene and 1 mg/ml of DEX	CI	100 μl	GelMA-MXene hydrogel not only exhibits good biocompatibility but also may improve cell conditions and enhance cell viability	Not mentioned	Guinea pigs weighed 200–230 g	(1) The GelMA + MXene + DEX group exhibited the least threshold shift at 4 and 8 kHz at day 28 postoperatively, and had a significant increase of SGNs in the basal, lower middle, and upper middle turns (2) In the basal turn, which is closest to the electrode implantation site, DEX and GelMA + MXene + DEX groups showed no detectable fibrosis, with a statistically significant reduction in fibrosis compared to the control groups	[77]
Micropump	The actuation force of the micropump is provided by expansion/shrinkage of a thermally phase-change material due to its melting/crystallization, uses 3D-printing technology, size about 8 × 8 × 3 mm^3^	Trans-RWM	50 nl/min, 20 min	A micropump prototype was implanted for 1 month without development of inflammation or infection	Not mentioned	CBA/CaJ mice, 2–4-month-old	There were systematic increases in distortion product threshold shifts during the 20-min perfusions, the mean shift was 15 dB for the most basal region	[215]

UC-MSCs, umbilical cord-mesenchymal stem cells; Exos, exosomes; RWM, round window membrane; PBS, phosphate-buffered solution; 3-MA, 3-methyladenine; SS-31, peptide d-Arg-2′,6′-dimethylTyr-Lys-Phe; EE, encapsulation efficiency; LE, loading efficiency; MC, minocycline; SS31-Lp, SS-31-modified liposome; SS31-Lp/MC, SS-31-modified liposome loaded with MC-treated group; GT, gentamicin sulfate; HCs, hair cells; AAV, adeno-associated virus; GFP, green fluorescent protein; EGFP, enhanced GFP; CMV, cytomegalovirus; PSCC, posterior semicircular canals; IDLR1, immunoglobulin-like domain-containing receptor 1; ABR, auditory brainstem response; Fer-1, ferrostatin-1; PLGA, poly(lactic-co-glycolic acid); LS19, polypeptide LS19; CS, chitosan; NPs, nanoparticles; DL, drug loading efficiency; GP, glycerophosphate; IT, intratympanic injection; CCK-8, Cell Counting Kit-8; PI, propidium iodide; OHC, outer hair cell; HGC, hexanoyl glycol; DEX, dexamethasone; CS; IHCs, inner HCs; MA-PDMS, methacrylated poly(dimethylsiloxane); GelMA, methacrylate gelatin; MXene, Ti3C2Tx MXene; CI, cochlear implantation; 3D, 3-dimensional

### Nanocarrier

Nanocarriers constitute an important part of biomedical engineering [118–125]. They have the advantages of improving drug stability and solubility, providing platforms for sustained and targeted drug delivery. They can be approximately divided into organic carriers, inorganic nanocarriers, and hybrid carriers based on their compositions [126]. According to the loading principle, nanocarriers are categorized into molecular loading, surface loading, matrix loading, and cavity loading systems [127]. To date, PLGA-based NPs, silica-based NPs (SNPs)/mesoporous silica-based NPs (MSNs), extracellular vesicles (EVs), exosomes, liposomes, and viruses are commonly used nanocarriers. Additionally, nanozymes have also demonstrated potential as drug carriers [128]. However, in the context of inner ear drug delivery, they are predominantly employed as therapeutic agents.

#### PLGA-based nanocarrier

PLGA, a copolymer of lactic and glycolic acid, exhibits good biocompatibility and biodegradability [129,130]. Zhang et al. [129] found that PLGA-based NPs crossed the RWM in a concentration-dependent manner via the transcellular pathway. However, the fluorescent integrity of the mimic drug in the RWM dropped at 60 min after administration, indicating low potential for long-term therapy. To address this problem, Ma et al. [131] loaded ferrostatin-1 (Fer-1) into a chitosan (CS)-PLGA-based, polypeptide LS19-coupled thermosensitive nanodelivery system, PLGA-CS-LS19-Fer-1-NPs (Fig. 6B). CS and glycerophosphate respectively empowered this system with stronger adhesion and thermosensitive properties.

#### SNPs/MSNs

SNPs/MSNs are widely used in the development of the biomedical area. SNPs/MSNs possess a large surface area, good mechanical stability, low cytotoxicity, and adjustable size [132–134]. The ordered porous structures of MSNs are beneficial for drug/gene loading and release control. Chen et al. [135] loaded transforming growth factor β1 (TGFβ1)–small interfering RNA (siRNA) into amino (-NH_2_) functionalized MSN (MSN-NH_2_), aimed at alleviating TGFβ secretion-induced fibrosis while patients receive CI. The TGFβ1 mRNA expression level was found to be 41.1% lower in MSN-NH_2_-siTGFβ1-transfected macrophages than that in the control group, indicating that this MSN-based inner ear delivery system is a promising option to mitigate CI-induced side effects.

#### EVs and exosomes

EVs are cell-secreted, natural carriers that formerly exist as intraluminal vesicles in multivesicular compartments [136]. Exosomes (round-shape carriers, diameter commonly ranging from 50 to 150 nm) are secreted after late endosomes/multivesicular compartments fuse with the plasma membrane [137]. Therefore, they possess low immunogenicity and are easily accessible. These properties have attracted much attention to explore exosomes in disease treatment [138,139]. Hao et al. [140] prepared neural progenitor cell-derived, microRNA-21-transfected exosomes, which recovered inflammation-related hearing loss at a concentration of 0.8 μg/μl. Inspired by cell therapy strategies [141], Liu et al. [142] demonstrated that mesenchymal stem cell-derived exosomes (Fig. 6C) could protect HCs from neomycin-induced damage by reducing ROS level.

#### Liposomes

Liposomes are artificial phospholipid bilayer vesicles similar to the structure of cell membranes and possess high drug loading capacity [143]. Hydrophilic drugs are commonly loaded in their aqueous core, and the lipid bilayer is employed for hydrophobic drugs [144]. Zou et al. [145] confirmed the good biocompatibility of liposomes, finding that no hearing impairments or inflammatory biological marker expression changes were observed after administration. To realize precise therapy, An et al. [146] prepared an LS19 (OHC targeting peptide)-coupled, liposome-based forskolin (FSK) delivery system. It was demonstrated that the hearing condition of mice presented significant improvement in the LS19-FSK-NP receiving group, while the control group performed severe hearing loss. Hou et al. [147] used HC mitochondria-targeting tetrapeptide SS-31 to modify liposomes; the optimized system could maintain the HC MET channel activity and would not result in alterations in gentamicin uptake.

### Virus carriers and gene therapy

Gene therapy has emerged as a pivotal treatment for SNHL. Since diverse biological entity-based materials have been rapidly developing in recent years, the selection of appropriate gene vectors is of paramount importance. AAV attracts great attention [7,33]. AAV-mediated SNHL treatment encompasses gene replacement, gene silencing, and gene editing. Gene replacement aims at providing functional proteins to recover gene mutation-induced protein loss [148]. Isgrig et al. [149] delivered *Ildr1* complementary DNA (cDNA) to the organ of Corti and the cochlear lateral wall of *Ildr1^w−/−^* mice, a human autosomal recessive hereditary hearing loss 42 (DFNB42) animal model, using AAV2.7m8 (targeting IHCs, OHCs, and supporting cells of the organ of Corti) and AAV8BP2 (targeting different types of cochlear lateral wall cells) vectors, respectively (Fig. 6Di). *Ildr1* cDNA delivery restored *Ildr1* gene expression, effectively enhanced tight junction network organization, and improved cochlear structural integrity. HC restoration primarily occurred in the OHCs, which exhibited a remarkable increase in survival rate (Fig. 6Dii). In *OTOF*-related gene therapy, given that the coding sequence of the OTOF gene (~6 kb) substantially exceeds the packaging capacity of AAV vectors (~ 4.7 kb), Qi et al. [150] designed a dual-AAV system to deliver the *OTOF* gene via 2 separate vectors. Hu et al. [33] specially developed 2 truncated myosin 15 (Myo15) promoters sufficiently compact for AAV packaging while enabling selective expression of human OTOF in HCs. Gene silencing has been demonstrated to function by down-regulating or inhibiting the expression of related genes, thereby preventing the accumulation of toxic proteins [148]. RNA interference (RNAi) is one of the major gene silencing techniques. Yoshimura et al. [151] proved the efficacy of RNAi-mediated gene silencing in a human Tmc1 deafness model—*Beethoven* heterozygous mice (*Tmc1^Bth/+^*). Compared with untreated mice (whose IHC hair bundles are apparently disorganized with fused stereocilia in the apical region), the AAV9 carrying transgene cassette of the microRNA Tmc1 (AAV9.miTmc1)-treated group preserved more IHC hair bundles and exhibited some morphologically normal hair bundles. Gene editing employs the clustered regularly interspaced short palindromic repeats (CRISPR)–CRISPR-associated systems (Cas), which enable specific base conversions [152]. This technique was proved effective in alleviating the *microRNA-96* 14C>A mutation in a mouse model. Ten weeks after injecting AAV serotype 2 (AAV2)–KKH variants of *Staphylococcus aureus* Cas9 (SaCas9-KKH)–single guide RNA (sgRNA), auditory brainstem response (ABR) results of the mice exhibited improvements at frequencies from 5.6 to 16 kHz. At 14 and 20 weeks after injection, the ABR thresholds showed improvements of 21 and 28 dB, respectively, ranging from 5.6- to 16-kHz frequencies as well. This suggests the efficacy of AAV2-mediated genome editing in both short-term and long-term hearing preservation [153]. Xue et al. [154] reported an AAV-delivered mini-dCas13X RNA editing system, mxABE, which promoted the conversion of TAG to TGG in a humanized *Otof* ^Q829X/Q829X^ mouse model. This editing strategy restored OTOF expression in nearly 100% of IHCs and rescued auditory function to near wild-type (WT) levels.

### Ultrasound-meditated microbubbles

USMB is a combination of ultrasound (US) and microbubbles (MBs), which has gained increasing attention in inner ear disorders therapy in recent years. USMB-mediated sonoporation has been demonstrated to promote the formation of tiny holes in the RWM, accompanied by minimal damage to the tissue, which subsequently enhances RWM permeability temporarily and augments the drug concentration delivered to the inner ear (Fig. 6E) [155,156]. In a guinea pig model, Shih et al. [157] observed that the inner ear perilymphatic fluorescence intensity of biotin-fluorescein 5-isothiocyanate (FITC) in the USMB group was notably higher than in the RW soaking group. Additionally, without US, MBs failed to elevate the inner ear drug concentration, confirming the critical role of US in enhancing RWM permeability. In another work, He et al. [158] identified USMB as a promising strategy for reversible opening of the BLB. As the primary hindrance in the systemic delivery route, opening the BLB greatly promoted the delivery of hydrophobic curcumin into the inner ear, which preserved nearly all HCs from 3 turns against cisplatin-induced damage. Overall, USMB is a potential strategy that promotes inner ear disease treatment.

### Microshotgun

In order to facilitate the crossing of biological barriers during inner ear drug delivery, Liang et al. [159] designed a gun-like, metal-free, slim-waist drug carrier. In this system, a microtube (thickness of about 15 μm, pore diameter of about 5 μm) functions as the barrel, NPs serve as the bullets, water acts as an ignitor, and solid dispersion acts as the fuel. Organic acids, basic carbonate salt, and water together yield the carbon dioxide gas, which enables NP initial acceleration and their penetration through the barrier’s outer layer. External magnetic fields continually propel the NPs to cross the inner layer tight junction. The cumulative amounts of NPs penetrating the RWM versus time (*M*−*t* curve) indicated that the crossing procedure comprised approximately 3 phases: an active ejection phase, a pseudo-stable state, and a stable state. Moreover, Yi et al. [160] designed a multiple-drug-loaded, tube-type microrobot based on a familiar mechanism. While exposed to US stimulation, perfluorohexane vaporization enables the microtube to behave like a microshotgun, promoting drug delivery to the inner ear. Furthermore, the proportion of drugs entering the inner ear is adjustable.

### Injectable hydrogels

Hydrogels are soft, 3-dimensionally crosslinked polymeric networks that have high water content and good biocompatibility, as well as adjustable mechanical strength and pore architecture [161–170]. These features allow hydrogels to be ideal drug carrier systems [171–173]. Among them, injectable hydrogels constitute an important subclass [174]. Beyond drug loading, they remain in a fluid or sol state before or during injection, and rapidly shift to a stable gel in situ under physiological conditions, thereby allowing a fit between the injectable hydrogel and irregular tissue structures [175]. Therefore, injectable hydrogels offer optimum drug delivery systems. When combined with a minimally invasive operation, these systems can effectively preserve delicate inner ear structures. To date, many types of injectable hydrogels, such as CS, GelMA, poloxamer, and silk fibroin (SF), have been widely used in inner ear drug delivery systems.

#### Chitosan

**Fig. 7.**
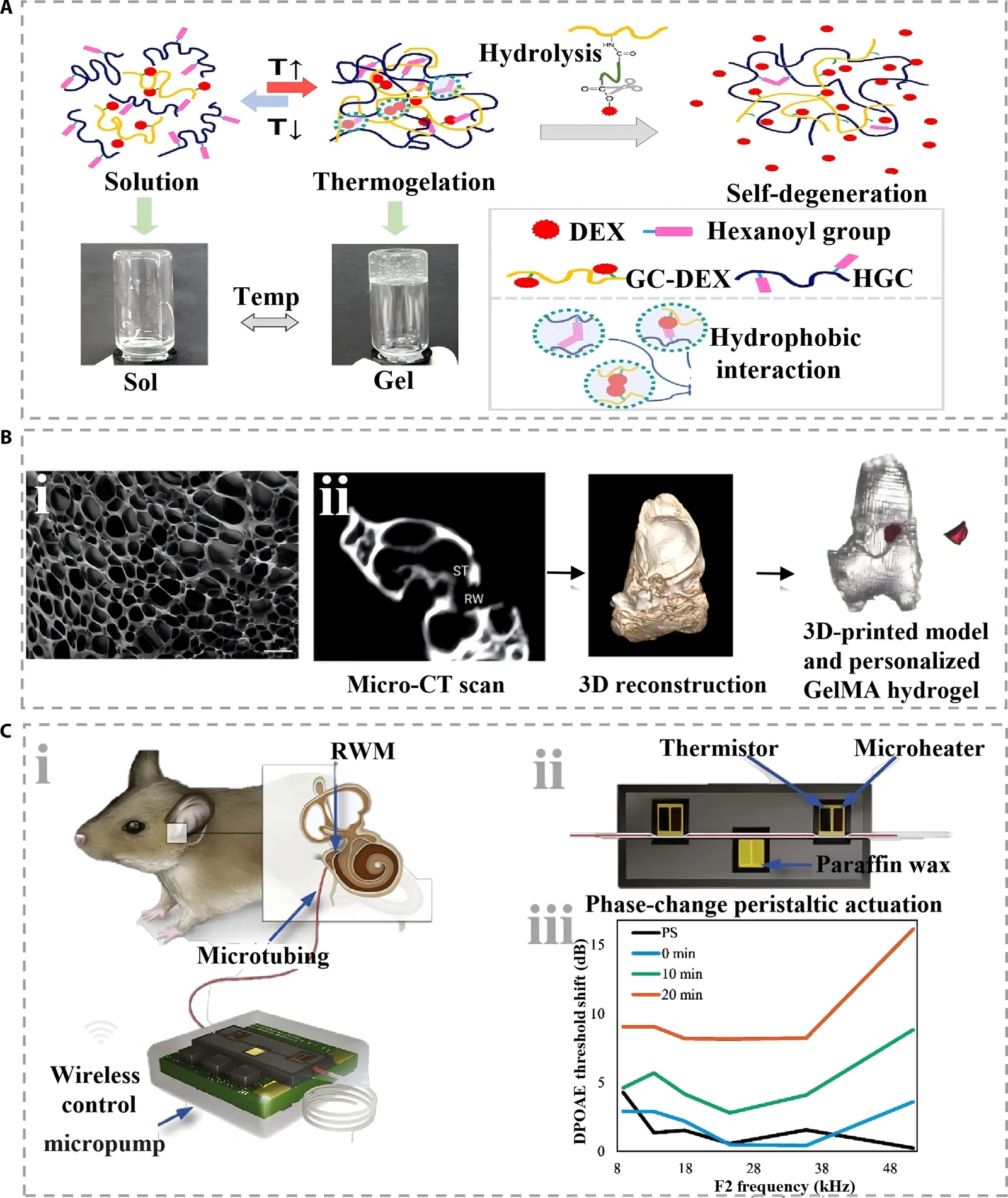
(A) Schematic illustration of the formation of self-degradable prodrug blend thermogel for intratympanic drug delivery. Reproduced with permission [175]. Copyright 2023, The Authors. (B) (i) SEM images of PGMA [200]. Copyright 2024, The Authors. (ii) Scheme of manufacturing personalized 3D mold for PGMA@NAM. Reproduced with permission [200]. Copyright 2024, Wiley-VCH. (C) (i) Schematic illustration of a nanoliter scale micropump for murine inner ear drug delivery. Reproduced with permission [215]. Copyright 2019, Elsevier. (ii) Cyclic phase-change actuation of the 3 chambers compressed the microtubing and drove peristalsis. Reproduced with permission [215]. Copyright 2019, Elsevier. (iii) Increasing mean DPOAE thresholds from different groups proved effectiveness of the inner ear micropump. Reproduced with permission [215]. Copyright 2019, Elsevier. Sol, solution; Gel, gelation; DEX, dexamethasone; GC-DEX, dexamethasone-conjugated glycol chitosan; HGC, hexanoyl glycol chitosan; GelMA, gelatin methacryloyl; PGMA, personalized porous GelMA; NAM, nicotinamide; PGMA@NAM, personalized NAM-encapsulated porous GelMA; 3D, 3-dimensional; CT, computerized tomography.

CS is a linear polysaccharide with good biocompatibility, biodegradability, ease of modification, and nontoxicity [176–180]. CS-based injectable hydrogels, including CS, glycol-CS (GC), and hexanoyl-GC (HGC), have attracted considerable interest as promising candidates for inner ear drug delivery systems. Saber et al. [181] verified the feasibility of CS as a carrier to deliver drugs across the RWM. Subsequent concentration-dependent inner ear damage in albino guinea pigs confirmed the effectiveness of neomycin delivery. Yu et al. [182] identified HGC as a promising thermogel formulation, as well as a solubilizing agent that facilitated DEX inner ear delivery. This thermosensitive system exhibited sustained stability over a period of 21 d and caused no cytotoxicity or inflammation. Phuc Le et al. [175] developed a polymer prodrug GC-DEX to initially enhance drug stability and solubility, and then mixed the GC-DEX with thermogelling polymer HGC, forming the thermosensitive, injectable inner ear drug delivery system HGC/GC-DEX (Fig. 7A). HGC/GC-DEX possesses long-lasting stability, enhancing hearing conditions 7 d after noise exposure.

#### Gelatin methacryloyl

GelMA is a derivative of gelatin with good biodegradability and biocompatibility, which is injectable and capable of forming hydrogels after polymerization [183–189]. However, a bulk of hydrogel in the middle ear cavity may impede acoustic transmission. Processing it into a drug-loaded microcarrier with microfluidic technique is an effective solution to achieve precise administration [190–197]. Wang et al. [198] prepared a GelMA-based hydrogel microsphere with a diameter of 40 μm, which served as an ideal inner ear drug delivery system for DEX-sodium phosphate (DSP) IT. It was confirmed that about 80% of the microspheres remained on the RWM for 1 week, thereby alleviating damage to the HCs and synaptic ribbons. Besides, considering the limited size of drug delivery systems, a targeted drug delivery system is urgently needed to minimize drug loss. Chen et al. [199] prepared a GelMA-microrobot that realized directional movement and targeted drug delivery via magnetic field control. A sustained and strong protective effect was shown in OHCs, which significantly facilitated hearing recovery. To increase the interfacial contact area between the material and the RWM, Feng et al. [200] novelly prepared a personalized porous GelMA (PGMA) (Fig. 7Bi). Beyond sustained and long-term drug release, nicotinamide-encapsulated PGMA (PGMA@NAM) achieved personalized in situ implantation with the assistance of micro-computed tomography (CT) and reconstruction of the RW niche (Fig. 7Bii). PGMA@NAM administration effectively protected mitochondria within IHCs and significantly alleviated hearing loss, and the ABR threshold measurement presented improvements after noise exposure.

#### Poloxamer

Poloxamer is a series of synthetic triblock copolymers with the structure poly(ethylene oxide)-b-poly(propylene oxide)-b-poly(ethylene oxide) (PEO-PPO-PEO), which shows temperature-dependent self-assembly property and good biocompatibility [201]. Among all poloxamers, poloxamer 407 has been widely reported for its application in inner ear drug delivery systems. Its cosolvent property allows the sustained release of DEX for about 96 h [202]. While solubility has improved, the sol/gel transition property remains a critical factor in ensuring ideal gelation time and suitable adherence of the poloxamer-based NAC delivery system [203]. In another study, the thermosensitive property prolonged drug retention time to 6 d in a poloxamer 407-based microemulsion gel (MEG) delivery system. Furthermore, along with optimization, this system presents 2 times higher cochlear drug absorption than other groups, thereby providing effective protection against cisplatin-induced damage [204]. Moreover, the combination of poloxamer 407 and USMB increased inner ear DEX delivery efficacy by 109.13% and 66.67% at 1 and 7 d post-administration, respectively [205].

#### Silk fibroin

SF is the primary component of naturally produced silk fiber, which is also an outstanding bioengineering polymer for its satisfying stability, mechanical strength, and good biocompatibility [206–208]. Owing to these properties, SF is considered a promising biomaterial in inner ear drug delivery systems [209]. For instance, Li et al. [210] developed a poly-_L_-lysine/SF coating that enables uniform distribution of DEX microcrystals. In addition to SF coating, Yu et al. [211] prepared a PEG-SF hydrogel for micronized DEX (mDEX) delivery, which can maintain perilymph DEX concentration over 100 ng/ml for 10 d, minimizing inflammation and systemic exposure. Xiong et al. [212] developed an SF-based hydrogel, which contained lipoic acid (LA) and DEX microcrystals. After injection, the hydrogel was shown to remain in the inner ear for 14 d and caused no obvious inflammation.

### Micropump

A micropump is an ideal reservoir to supply sufficient drugs and achieve precise dose control. Kim et al. [213] developed an advanced reciprocating micropump system that achieves zero net volume liquid transfer, with a reciprocating volume of about 1.5 μl. The operating cycle is initiated by priming the pump with biocompatible solution, which is subsequently infused into the cochlea via the cannula. Following infusion, the device withdraws a portion of the perilymph–drug mixture from the tip of the cannula, as the remaining drug continuously diffuses into the cochlear tissues. For preparing the next administration, diluted drug is moved to the reservoir end, while fresh drug is loaded from the opposite side [213]. Borenstein and coworkers [214] verified the feasibility of their controlled, automated drug delivery reciprocating micropump by dinitroquinoxaline-2,3-dione (DNQX) in vivo infusion. Guinea pig compound action potential (CAP) threshold elevation at all frequencies suggests the efficacy of the developed micropump. Forouzandeh et al. [215] novelly reported a nanoliter-scale micropump that achieves pumping by the expansion and contraction of thermal phase-change material, which can sequentially compress the microtube. Resistive heaters and thermistors were arranged in line to provide templates with chambers and microtube grooves, and thermal phase-change paraffin was directly located in those chambers (Fig. 7Ci and ii). To test the feasibility of this micropump as an inner ear drug delivery system, the ototoxic drug sodium salicylate was delivered at a speed of 50 nl/min for 20 min. Systematically increasing distortion product otoacoustic emission (DPOAE) shift thresholds suggested the effectiveness of this drug delivery system (Fig. 7Ciii). Moreover, this wirelessly controlled micropump exhibited good biocompatibility, indicating its potential in becoming a hopeful platform for inner ear therapy. Nonimplantable micropumps can also assist in the treatment of hearing loss. During gene delivery, the total injection volume of viral vectors is typically only 1.0 μl [151], and artificial administration may result in reagent loss or even structural damage to the inner ear. Furthermore, precise control over the injection rate is difficult to achieve manually. CMA 102 microdialysis pump was reported to offer a uniform solution at a speed of 10 nl/s [216], thereby ensuring accurate delivery of therapeutic agents.

## Ongoing Clinical Trials

The rapid development of engineered inner ear drug delivery systems has accelerated their translation toward clinical application. Numerous clinical trials investigating drug delivery strategies have been completed or are currently ongoing. Discussing about the anti-inflammatory therapeutic agents, the DEX formulation OTO-104 was demonstrated to be effective in alleviating vertigo associated with Ménière’s disease (NCT02717442, NCT02612337, NCT03664674), but only one of these trials showed statistical significance [217]. Ebselen (SPI-1005), a novel drug, has shown potential in reducing temporary threshold shifts and preventing NIHL progression [218]. Beyond anti-inflammatory agents, several neuroprotective, otoprotective, and regenerative therapeutics have also demonstrated promising outcomes. Intratympanic administration of a 0.87 mg/ml gel-formulated *N*-methyl-d-aspartic acid (NMDA) receptor antagonist, AM-101, has been shown to alleviate tinnitus (NCT01803646) [219]. ORC-13661, a high-affinity permeant blocker of the OHC MET channel derived from PROTO-1, has been shown to reduce the toxicity of aminoglycoside antibiotics [220]. A phase II trial is currently investigating the efficacy of ORC-13661 in preventing hearing loss in patients with nontuberculous mycobacteria infection undergoing amikacin therapy (NCT05730283). Regenerative therapeutics FX-322, the combination of CHIR99021 and valproic acid, has been shown to regenerate mammalian cochlear HCs ex vivo. IT of FX-322 has shown an approximately 10-dB improvement at 8 kHz [221].

Regarding gene therapy, several encouraging advances have recently been reported. The largest global clinical trial on *OTOF*-related deafness has recently yielded exciting results. Jiang et al. [4] reported that AAV serotype 1 carrying a human *OTOF* coding transgene (AAV1-hOTOF) presented long-term therapeutic effects among patients aged 0.8 to 32.3 years. Ninety percent of the patients presented hearing recovery during the follow-up 2.5 years; some of the patients could perceive conversational speech, library-level sounds, and even whisper. In 2026, the U.S. Food and Drug Administration (FDA) approved Otarmeni (lunsotogene parvec-cwha), the first dual-AAV-based gene therapy. After therapy, 80% of the 24 patients (aged from 10 months to 16 years) experienced hearing recovery [5].

Despite these promising advances, clinical outcomes have often been inconsistent or disappointing. The discrepancy between preclinical findings and clinical outcomes remains a critical challenge. Rational participant selection criteria are fundamental to clinical trials, as real clinical patients are more heterogeneous and exhibit greater complexity in terms of age, comorbidities, and medication history. Even when trials are carefully designed, many of them are terminated at phase I/II due to safety concerns, strong placebo effects, and potential immune responses, as well as poor patient adherence during long-term treatment. Furthermore, large-scale manufacturing, quality control, regulatory approval, and clinical implementation collectively pose important challenges to the successful translation of therapeutic strategies into clinical practice.

## Challenges and Outlook

Hearing loss has emerged as a global health concern, driving a growing demand for effective therapeutic strategies and stimulating advances in both drug delivery routes and inner ear drug delivery systems. Therefore, a comprehensive understanding of inner ear drug delivery is essential for providing patients with convenient and highly efficacious treatments. This review summarizes several drug delivery pathways, commonly used therapeutic agents for inner ear disorders, and recently developed bioengineered delivery systems while also addressing current challenges and limitations in treatment.

Over recent decades, substantial progress has been achieved in the field of inner ear drug delivery. However, several challenges continue to limit the selection of appropriate administration routes. The complex and delicate structure of the inner ear, together with the presence of the BLB, restricts systemic delivery. Considering the BLB heterogeneity in molecular expression, cellular composition, and spatial structure, it is challenging for therapeutic agents to achieve their target site at sufficient concentrations. Shi et al. [222] reported low-density lipoprotein receptor-related protein 1 as a promising target for opening the BLB. US-mediated techniques are supposed to enhance the BLB permeabilization by widening its tight junctions [158]. Precise administration routes, including trans-tympanic and intracochlear administration, have also been widely studied to bypass the BLB. However, perilymph pharmacokinetics may lead to uneven drug distribution. For instance, small lipophilic molecules would be eliminated rapidly. As a result, it could only treat basal cochlear regions (high-frequency) of the cochlea and cannot reach the apical regions [223]. Limited RWM permeability, eustachian tube clearance, and limited drug retention time further constrain therapeutic efficacy. Therefore, in addition to selecting appropriate routes of administration, increasing attention has been directed toward the innovation and optimization of drug carriers. NPs are functionalized with targeting ligands or formulated with stimuli-responsive properties (e.g., ROS-responsive, pH-responsive, or thermosensitive) to cross the biological barriers, enabling controlled and sustained drug release at specific sites. Targeting ligands enhance the RWM permeability to NPs and promote cellular uptake [61]. ROS-responsive NPs could cross the RWM via endocytic pathways, enter the perilymph of the basilar membrane, and diffuse longitudinally and laterally. After entering the inner ear, the pH-responsive drug delivery system releases therapeutic agents in response to the pH alterations in the inner ear [105]. Microshotgun techniques have been shown to enhance drug transport across biological barriers via gas generating reactions of self-contained chemicals [159]. Injectable hydrogels are suitable carriers for drugs as well. Their thermosensitive characteristics, along with prolonged retention time, effectively address the limitation of Eustachian tube clearance. As some patients require long-term treatment, or wish to avoid repeated invasive procedures, implantable micropump systems can enable continuous and controlled drug administration. Overall, these advances have facilitated the development of multimodal therapeutic strategies to achieve improved therapeutic effects. For example, drug-loaded hydrogel electrode coatings have been shown to effectively mitigate CI-associated fibrosis [76]. In gene therapy, a DEX gene co-delivery NP has been shown to enhance anti-inflammatory effects and improve gene expression [224]. As AAVs may induce immune responses in mammals, and their limited packaging capacity may compromise therapeutic efficacy, helper-dependent adenoviral vectors were studied to provide larger packaging capacity (36 kb) [225,226]. Collectively, these advances provide promising platforms for the more precise design of inner ear drug delivery systems.

 Before applying to humans, these emerging strategies are often first evaluated in rodent models. However, notable anatomical differences exist between humans and rodents (Table 2) [227]. First, the smaller TM size and tympanic cavity volume in rodents result in a higher relative drug concentration following intratympanic administration, facilitating rapid diffusion toward the RW. Second, rodents typically exhibit a relatively larger RWM, promoting efficient drug entry into the inner ear. Third, the small cochlear volume in rodents allows for rapid equilibration and relatively uniform drug distribution. Besides differences between humans and rodents, patient-specific factors should also be taken into consideration. Age is an important determinant of treatment strategy. Anesthetic risk should be considered, especially for pediatric and geriatric patients. Additionally, age-related changes may require highly precise administration in elderly patients. As noted above, pathological heterogeneity across inner ear disorders necessitates distinct therapeutic targets and strategies. Moreover, a thorough understanding of concomitant medications is critical. Patients receiving anticoagulant or antiplatelet therapy may require minimally invasive approaches to avoid bleeding risks, whereas those undergoing ototoxic chemotherapy may benefit from localized delivery strategies that minimize interference with systemic treatment.

**Table 2.** Anatomy differences between human and rodents [227]

	Human (weighed of 70 kg, *n* = 4)	Guinea pig (weighed of 400 g, *n* = 3)	Rat (weighed of 30 g, *n* = 4)
TM area (mm²)	91	34	5.4
Anteroposterior TM diameter (mm)	10.2 ± 0.66	6.8 ± 0.15	1.9 ± 0.10
Tympanic cavity volume (mm^3^)	625	287	6.4
RW diameter (mm)	1.6 ± 0.07	0.8 ± 0.05	0.3 ± 0.02
Cochlea volume (mm^3^)	78	11	1.08

TM, tympanic membrane; RW, round window

Overall, in order to achieve precise therapy, we need to take both timely diagnosis and personalized administration into consideration. Hearing loss is typically assessed using audiologic testing, with pure-tone audiometry evaluating tone detection across a range of frequencies and indicating the hearing loss category. Additionally, genetic testing is crucial for newborn hearing screening. Next-generation sequencing (NGS) has detected *GJB2*, *SLC26A4*, *mtDNA*, and *GJB3* genes and found that 195 of 1907 (10.22%) are carriers of the deafness gene, which offers the possibility for timely therapy [228]. Regarding imaging studies, magnetic resonance imaging (MRI) may achieve visualization of endolymphatic hydrops in Ménière’s disease patients [49]. Considering the selection of therapeutic agents, a clear understanding of weather the patients have ototoxicity medication history or operation history is of great importance. Corticosteroid intervention within 2 weeks and intratympanic steroid therapy are recommended once symptoms appear [229]. Nevertheless, the low solubility of DEX limits its administration at low concentrations, and DSP-loaded GelMA-NPs can help overcome this limitation [198]. Moreover, limited residence time and reduced therapeutic exposure require repeated administration or hydrogel-based sustained-release systems.

Since these novel delivery systems move toward clinical translation, alignment with regulatory requirements is increasingly important. Regarding clinical trial application (CTA), different countries and regions have established their own regulatory standards. For instance, investigational new drug (IND) submissions are requiring support from a comprehensive preclinical evidence package. Such a package should include detailed animal pharmacology and toxicology studies, manufacturing information, and clinical protocols and investigator information [230]. Therefore, a clear understanding of the delivery system classification of these regulatory requirements is essential to facilitate successful clinical translation. Regarding the delivery system itself, for instance, when therapeutic agents are combined with intracochlear devices, it could be seen as a combination product. Federal Register (FR) has clearly classified different types of products [231]. In inner ear drug delivery systems, there are basically drug–device combinations (DEX-coating cochlear implants) and biologic–device combinations (stem cell-coated electrode) [232,233]. However, the cochlea is a surgically inaccessible end-organ, which limits repeated biopsy and direct histological evaluation. FDA has already classified CI into class III, reflecting their high-risk nature and the requirement for premarket approval [234]. This indicates that the combination product would go through strict assessment premarketing. In addition, the Office of Combination Products (OCP) designates the appropriate FDA medical product center to conduct the review of combination products and ensures the continued safety and quality of combination products through oversight of post-market activities [235].

Overall, several key considerations should guide the future development of inner ear drug delivery systems. Biocompatibility and stability remain fundamental requirements for diverse delivery platforms. Prodrug strategies offer potential for improved therapeutic efficacy, while controlled release mechanisms must be optimized to ensure sustained drug delivery rather than an initial burst release. Furthermore, the translatability of animal studies to human applications requires careful consideration. Addressing these challenges will be crucial for developing the next generation of innovative inner ear drug delivery systems, particularly through the integration of multimodal strategies and precision medicine approaches.
